# Transcranial Magnetic Stimulation for Long-Term Smoking Cessation: Preliminary Examination of Delay Discounting as a Therapeutic Target and the Effects of Intensity and Duration

**DOI:** 10.3389/fnhum.2022.920383

**Published:** 2022-07-05

**Authors:** Alina Shevorykin, Ellen Carl, Martin C. Mahoney, Colleen A. Hanlon, Amylynn Liskiewicz, Cheryl Rivard, Ronald Alberico, Ahmed Belal, Lindsey Bensch, Darian Vantucci, Hannah Thorner, Matthew Marion, Warren K. Bickel, Christine E. Sheffer

**Affiliations:** ^1^Roswell Park Comprehensive Cancer Center, Buffalo, NY, United States; ^2^Wake Forest School of Medicine, Winston-Salem, NC, United States; ^3^Fralin Biomedical Research Institute at Virginia Tech Carilion, Roanoke, VA, United States

**Keywords:** transcranial magnetic stimulation, smoking cessation, tobacco dependence treatment, delay discounting, self-regulation, brain stimulation

## Abstract

**Background:**

Repetitive transcranial magnetic stimulation (rTMS) is a novel treatment for smoking cessation and delay discounting rate is novel therapeutic target. Research to determine optimal therapeutic targets and dosing parameters for long-term smoking cessation is needed. Due to potential biases and confounds introduced by the COVID-19 pandemic, we report preliminary results from an ongoing study among participants who reached study end prior to the pandemic.

**Methods:**

In a 3 × 2 randomized factorial design, participants (*n* = 23) received 900 pulses of 20 Hz rTMS to the left dorsolateral prefrontal cortex (PFC) in one of three Durations (8, 12, or 16 days of stimulation) and two Intensities (1 or 2 sessions per day). We examined direction and magnitude of the effect sizes on latency to relapse, 6-month point-prevalence abstinence rates, research burden, and delay discounting rates.

**Results:**

A large effect size was found for Duration and a medium for Intensity for latency to relapse. Increasing Duration increased the odds of abstinence 7–8-fold while increasing Intensity doubled the odds of abstinence. A large effect size was found for Duration, a small for Intensity for delay discounting rate. Increasing Duration and Intensity had a small effect on participant burden.

**Conclusion:**

Findings provide preliminary support for delay discounting as a therapeutic target and for increasing Duration and Intensity to achieve larger effect sizes for long-term smoking cessation and will provide a pre-pandemic comparison for data collected during the pandemic.

**Clinical Trial Registration:**

[www.ClinicalTrials.gov], identifier [NCT03865472].

## Introduction

*“Making progress on longstanding challenges requires a different lens and a new approach.”*

Ayanna Pressley.

Over one-half of individuals who smoke cigarettes in the US attempt to quit every year, but over 90% rapidly reverse this decision, choosing the immediate reward of smoking over the long-term benefits of quitting (Babb et al., 2017). Despite the increased availability of evidence-based behavioral and pharmacological treatments for cigarette smoking, less than one-third of cigarette smokers use them (Babb et al., 2017; Office on Smoking and United States Public Health Service Office of the Surgeon General, and National Center for Chronic Disease Prevention and Health Promotion (US) Office on Smoking and Health, 2020). Negative attitudes about taking medications for smoking cessation are a commonly endorsed barrier to using pharmacological treatments (Mooney et al., 2006; Gross et al., 2008; Morphett et al., 2015; Smith et al., 2015). Novel, non-pharmacological treatment approaches have the potential to provide cigarette smokers with more smoking cessation treatment options.

Tremendous progress has been made in the development of brain stimulation techniques to support smoking cessation (Ekhtiari et al., 2019). High frequency (HF) (rTMS) is a non-invasive brain stimulation technique that can selectively modulate neuronal plasticity (Ekhtiari et al., 2019). Using a variety of different coil configurations, rTMS generates brief focal electromagnetic pulses that penetrate the skull to stimulate brain regions via localized axonal depolarization (Fitzgerald et al., 2006; Thut and Pascual-Leone, 2010). rTMS coil selection is based on the need for stimulation depth and focality (Lu and Ueno, 2017). HF rTMS using an H4-coil was recently cleared by the Federal Drug Administration (K200957) for short-term smoking cessation (Zangen et al., 2021). The H4-coil stimulates broad swaths of the (PFC) and insula (Fiocchi et al., 2018). The conventional figure of 8 coil delivers stimulation with more focality than the H4 coil (i.e., targets with more specificity) (Lu and Ueno, 2017). HF rTMS of the left dorsolateral PFC (dlPFC) using a figure of 8 coil is emerging as a novel non-pharmacological treatment approach for long-term smoking cessation (Ekhtiari et al., 2019).

The Competing Neurobehavioral Decisions Systems (CNDS) Model is a broad, fundamental framework grounded in neuroeconomics and dual processing theory (Mukherjee, 2010). The CNDS Model describes the general neurobiological underpinnings involved with making far-sighted decisions about one’s health (e.g., maintaining abstinence from smoking) in the context of immediately rewarding, though less healthy choices (e.g., continued smoking) (Bickel et al., 2007; McClure and Bickel, 2014). The Model posits that these decisions are broadly driven by the interaction between two functional neurobiological networks: the executive function network, embodied in the PFC; and the impulsive network, embodied in the limbic and paralimbic regions of the brain (Bickel et al., 2014, 2016; Hanlon et al., 2015). The balance of activity in these two networks shapes reward-related decision-making (Hanlon et al., 2015). Greater activity in the PFC is linked with a higher likelihood of more prudent decision-making, even in the context of temptation (McClure et al., 2007; MacKillop et al., 2012). However, chronic nicotine administration can significantly impact reward-related decision-making (Koob, 2008a,b) and over time, the balance and functioning of these networks can become dysregulated, resulting in significant deficits in executive function neural processing (Ernst et al., 2001; Xu et al., 2005; McClure and Bickel, 2014; Koob et al., 2014). Dysfunction or hypo-activation of the executive function network contributes to undervaluation of the long-term rewards from cessation (Hanlon et al., 2015), adding to the behavioral and psychosocial challenges of achieving long-term abstinence from cigarettes.

Most individuals prefer immediate rewards because reinforcement loses value the longer one waits to receive it, but individuals demonstrate considerable variability in these preferences. Delay discounting rate represents the degree to which individuals discount the value of a reward as a function of time to receipt (Kirby, 1997; Odum, 2011; Commons et al., 2013). Delay discounting rate is considered a transdiagnostic biological marker for the relative balance between the executive and impulsive networks consistent with the CNDS model (Bickel et al., 2012, 2019), and is a well-established prognostic factor for smoking cessation treatment outcomes (Sheffer et al., 2012, 2014; Coughlin et al., 2020). Importantly, delay discounting rates are malleable, with decreases associated with improved health behaviors (Koffarnus et al., 2013; Bickel et al., 2014; Rung and Madden, 2018).

The frontolimbic balance outlined by the CNDS Model and its application to cigarette smoking, however, must be viewed as a general framework within the context of the multiple complex neurobiological, psychological, affective, environmental, socio-cultural, and evolutionary factors that contribute to the development and maintenance of dysfunctional human decision-making, a review of which is outside the scope of this manuscript (Alcaro et al., 2021). For instance, the well-established role of classical and operant conditioning in decision-making is optimized by the mesolimbic dopaminergic (MS DA) system (Robinson and Berridge, 2001, 2003, 2008; Salamone and Correa, 2002; Everitt and Robbins, 2005; Alcaro et al., 2007) and contributes significantly to the development and maintenance of the imbalance described by the CNDS Model. A significant body of research also shows that the SEEKING or exploring drive is neurologically foundational to all appetitively motivated behaviors (Alcaro et al., 2021). Addiction likely reflects dysfunction of the SEEKING drive, linked with the MS DA system and consistent with results predicted by the CNDS Model (Alcaro et al., 2021). In addition, delay discounting is one of many potential transdiagnostic dimensions that are relevant to reward dysfunction. Anhedonia, defined as the inability to feel pleasure, is a transdiagnostic dimension present in a wide variety of mental health and substance use disorders (Spano et al., 2019). As a symptom of abstinence from many substances, anhedonia can prevent adequate reinforcement from non-substance related reinforcers (Garfield et al., 2014).

The dlPFC, a functional node in the PFC, has a significant role in executive function and the controlled response inhibition associated with drug-related craving and self-regulation (Ernst et al., 2001; Brody et al., 2002; McBride et al., 2006). The proposed mechanism by which HF rTMS of the left dlPFC supports smoking cessation is by increasing neuronal activity and plasticity in the left dlPFC, thereby improving executive functions mediated by the dlPFC. Preliminary evidence indicates that the approach is feasible and can reduce delay discounting rates and increase short-term latency to relapse, abstinence rates, and uptake of psychoeducational material (Sheffer et al., 2013, 2018; Ekhtiari et al., 2019). Prior to conducting a large-scale study of efficacy, however, research is needed to determine the optimal dosing strategies to achieve long-term abstinence (i.e., 6 months or more).

The parent project from which this study is derived is an ongoing 5-year study aimed to determine the optimal dosing strategies of rTMS of the left dlPFC for long-term smoking cessation (Carl et al., 2020). This study employs a fully crossed, 3 × 2 × 2 randomized double-blinded factorial design, where Duration is defined as 8, 12, and 16 days of stimulation, Intensity is a number of pulses per day (900 in one session vs. 1,800 in two sessions), and participants are randomized to active/sham conditions.

The onset of the COVID-19 pandemic paused all in-person study-related activities in the parent study in late March of 2020. Once the study resumed, multiple factors associated with COVID-19 introduced possible biases and confounds on recruitment, retention, and outcomes. These factors include the need to use different recruitment strategies (social media vs. flyers in the community), increased participant burden due to safety precautions, concerns about physical distancing, and COVID-19 stress-related effects on engagement and outcomes. Thus, we report preliminary results from participants who reached study end prior to the COVID-19 outbreak in Western New York.

Our primary goal was to examine the effects of increasing rTMS Duration and Intensity of active stimulation on latency to relapse, 6-month point prevalence abstinence rates, participant burden, and delay discounting rates among participants who reached study end. The hypotheses were consistent with the parent study (see above). Given the preliminary nature of this study, the focus on pre-COVID participation, the 3–1 active/sham randomization in the parent study, and the limited sample size, we included only active participants, limited comparisons, and focused on effect sizes. Statistical significance, while reported, must be viewed with caution in this context, however, multiple statistical approaches were employed to establish consistency among the findings.

## Materials and Methods

### Participants

We recruited right-handed adults (age 18–65) who smoked 6–25 cigarettes daily and who were motivated to quit smoking. Participants were required to pass a 12-panel urine drug test, a pregnancy test, the TMS Adult Safety and Screening Questionnaire (TASS) (Rossi et al., 2011), claustrophobia screen to assess the ability to undergo a closed MRI of the head (Carl et al., 2020). Participants were recruited using flyers in the community, print advertisement, and social media.

### Design

This study is a fully crossed 3 × 2 randomized factorial design. The two factors were Duration (8, 12, or 16 days of stimulation) and Intensity (900 or 1,800 pulses per day). Although only those participants who received active stimulation were included in the analyses, all participants and technicians were blinded to active/sham condition. Participants were followed for 6 months after the quit date. Daily number of cigarettes smoked per day was collected every 2 weeks. Exhaled carbon monoxide (CO) level was assessed at each in-person outcome assessment (4-, 8-, 12-, 18-, and 24-weeks after the quit date). Given the preliminary nature of this study, the focus on pre-COVID participation, the 3–1 active/sham randomization in the parent study, and the limited sample size, only those participants who received active stimulation and reached study end prior to April 4, 2020 were included in this study.

### Procedure

Participants were screened over the telephone and invited to an in-person interview during which urine drug and pregnancy tests were administered. After informed consent, participants completed baseline assessments and were scheduled for an MRI. Prior to the MRI, the International 10-10 Electrode System was used to place a vitamin E capsule at the AF3 electrode position as a fiducial marker on the image. AF3 was chosen because the cognitive functions of interest are located in the anterior region of the dlPFC (Cieslik et al., 2012). The MRI was uploaded into the neuronavigation system with the fiducial marker readily apparent on the image. The MRI was also used to identify brain abnormalities that might impact participant safety. Eligible participants were randomized and scheduled for quit counseling, a quit date, and rTMS sessions.

The quit day was the day immediately prior to the first rTMS session. Participants were provided 30 min of brief structured cognitive behavioral counseling over the telephone 2 days prior to the quit date. Participants were required to abstain from smoking for at least 24 h prior to the first stimulation session, as evidenced by an expired breath CO level of < 10 ppm (SRNT Subcommittee on Biochemical Verification, 2002). Immediately prior to initiating rTMS, participants were randomized using permuted block randomization stratified by high or low nicotine dependence level [FTND; high (≥5) or low (<5)] (Heatherton et al., 1991).

TMS power was tailored to the Motor Threshold (MT), which was defined as the minimum stimulation power required to elicit a motor evoked potential of 50 μV from the abductor pollicis brevis (APB) in 3 of 6 trials. Each rTMS session provided 900 pulses of 20 Hz rTMS to the left dlPFC at 110% of the MT. Magstim Super RAPID^2^ PLUS1 System with Magstim 70 mm Double Air Film Active Figure of 8 Coil was used. The Brainsight Neuronavigational system (Rouge Research, Inc.) was used to track the placement of the coil in real time with respect to an MRI-derived image. Pulses were delivered in 45 20-pulse trains of 1 s duration with an inter-train interval of 20 s. Stimulation time was 16 min. Participants read psychoeducational materials (Forever Free^®^ self-help booklets) during the first 8 stimulation sessions. Participants were compensated $20 after each in-person visit, a weekly $50 bonus for completing all scheduled rTMS sessions, and $100 bonus for completing all five outcome assessments.

### Bioethics

The study was approved by the Institutional Review Board of Roswell Park Comprehensive Cancer Center (#I-65718). Informed consent was obtained from all participants.

### Measures

Demographic information collected at baseline included age, sex, race, ethnicity, partnered status, education, and household income. Other measures included the Fagerström Test for Nicotine Dependence (FTND) (Heatherton et al., 1991; Fagerstrom, 2012) and other clinical factors such as impulsivity measured by Barratt Impulsiveness Scale (Patton et al., 1995). Delay discounting and participant research burden were assessed at baseline and at each outcome assessment point. Primary outcomes included latency to relapse (number of days to relapse), 6-month point prevalence abstinence rates, participant research burden, and delay discounting rates.

Delay discounting rates were assessed using the 5-trial adjusting delay task for $100 and $1,000 magnitudes (Koffarnus and Bickel, 2014). During this task, participants were presented with a choice between two hypothetical monetary amounts ($100 vs. $50 in the $100 condition and $1,000 vs. $500 in the $1,000 condition). In each of the seven choice presentations, the smaller amount was available immediately, and the higher amount available at a discrete delay, beginning with 3 weeks. Based on the participant’s choice, delay either increased (when participants select the delayed option) or decreased (when participants select the immediate option). Participants made this choice for five trials, resulting in potential *k*-values which were subsequently log transformed into ln*k*.

Latency to relapse and point prevalence abstinence were assessed using the Timeline Follow Back procedure (TLFB) every 2 weeks (Sobell and Sobell, 1992; Brown et al., 1998) by telephone and during the in-person outcome assessments. Relapse was defined as 7 consecutive days of any cigarette smoking (Hughes et al., 2003). CO in exhaled breath of ≤ 5 ppm as measured by the Micro + Smokerlyzer (Covita, Inc.) was considered abstinent from cigarette smoking (SRNT Subcommittee on Biochemical Verification, 2002).

Participant research burden was assessed with the 21-item Perceived Research Burden Assessment (PeRBA) (Lingler et al., 2014). Total scores range from 21 to 105, with lower scores reflecting lower participant burden.

### Statistical Analyses

Descriptive analyses were used to characterize the sample. Because the aim was to examine the direction and strength of effect sizes, the primary tests were Cohen’s d, partial eta squared (η^2^), Cramer’s Phi squared (Φ^2^), and odds ratios (OR), as appropriate (Rhea, 2004; Le and Marcus, 2012; Tomczak and Tomczak, 2014; Enzmann, 2015; Kim, 2015). Cohen’s d provides a standardized difference between two means by expressing the difference in units of standard deviation. With Cohen’s d, a small effect size is ∼0.2, medium is ∼0.5, and large is ∼0.8 or greater. Partial η^2^ measures the proportion of variance explained by the dependent variable attributable to the independent variable. With η^2^, a small effect size is ∼ 0.01, medium is ∼0.06, and large is ∼0.14 or greater. Cramer’s Phi (Φ) reflects the strength of the association between two variables, and when squared, reflects how much variance is accounted for by the association. With Φ^2^, a small effect size is ∼0.01, medium is ∼0.09, and large is ∼0.25 or greater (Maher et al., 2013; Ialongo, 2016). OR reflect the direction and strength of the effect relative to the comparison group. Hazard ratios, chi square, confidence intervals, F-statistics, and *p*-values are reported as appropriate, but given small sample size, must be viewed with caution.

Cox proportional hazard (CPH) models were used to examine the effects of Duration and Intensity on latency to relapse. Days to relapse were right-censored. Right censoring was defined as participants who did not relapse while under observation, either because they maintained abstinence to the end of the study period or were lost to follow-up. Participants were considered abstinent at least as long as they were observed to have been abstinent. Cohen’s d was calculated for CPH results using the formula: *d* = *ln(HR)x✓(6/π)* (Azuero, 2016).

Binary logistic regression models were used to examine the effects of Duration and Intensity on 6-month/7-day point prevalence abstinence rates. Missing data was imputed as smoking for the Intention to treat analysis (ITT). Missing data was excluded for complete case analysis (CCA). For Duration, 8 days was used as the comparison group. For intensity, 1 session per day (900 pulses) was used as the comparison group. OR and confidence intervals are reported.

The analysis of delayed discounting rate and PeRBA was conducted in two ways: (1) Repeated measures multivariate analysis of variance (MANOVA) was used to examine main effects, and (2) Generalized estimating equations (GEE) was used to examine rate of change across time. Dependent variables were discounting (ln*k*) of $100 and $1,000 magnitudes, and the total score of the PeRBA. Time was entered as a within-subject factor, with six timepoints: baseline and 5 outcome assessments (4-, 8-, 12-, 16-, and 24-weeks after the first rTMS session). The Bonferroni adjustment was used to control for multiple comparisons. When Mauchly’s test statistic was significant, Greenhouse–Geisser correction was applied. Partial η^2^ was calculated for MANOVA results using the formula: η^2^ = SS_effect_/SS_total_ (Lakens, 2013). Cramer’s Phi (Φ) was calculated for GEE results using the formula: Φ = ✓(χ^2^/N); Φ^2^ was calculated to show shared variance (Phi/Cramer’s Phi; Sharpe, 2015).

## Results

### Participant Characteristics

Prior to pausing the parent project, *n* = 23 participants in the active condition reached study end. Participants were primarily middle-aged (*M* = 50.78, SD = 10.96). About 30% identified as non-white and 70% as women. Participants included a high proportion of individuals of lower income and were diverse in terms of employment status. Nearly 80% were Medicaid and/or Medicare beneficiaries, over half had household incomes less than $25,000 per year, and 40% did not attend college. Participants were highly dependent on smoking. Most began smoking as adolescents, and over half had not made a quit attempt in the past year. Participants were moderately confident in their ability to quit smoking and maintained relatively high levels of motivation to quit throughout the study. Baseline levels of overall impulsivity were moderate and remained steady throughout the study (see Table 1 and Supplementary Table 1).

**TABLE 1:** Participant (*n* = 23) characteristics at baseline.

Variable	Range or categories	M (SD) or% (n)
Age	20–64 years	50.78 (10.96)
Sex	Female	69.6% (16)
Race	White or Caucasian	69.6% (16)
	Black or African American	13% (3)
	Other	17.3% (4)
Ethnicity	Non-Hispanic	87% (20)
Partnered status	Un-partnered	52.2% (12)
Annual household income	<$10,000	17.4% (4)
	$10,000–$24,999	39.1% (9)
	$25,000–$74,999	39.1% (9)
	>$75,000	4.3% (1)
Highest education level	High school	39.1% (9)
	College	47.8% (11)
	Graduate school	13% (3)
Employment status	Full time	30.4% (7)
	Part-time	13% (3)
	Retired	13% (3)
	Disabled	8.7% (2)
	Unemployed	17.4% (4)
	Homemaker	17.4% (4)
Health insurance status	Medicare and/or Medicaid	78.2% (18)
	Private	17.4% (4)
	None	4.3% (1)
Cigarettes per day	6–25	14 (5.510)
Categories	6–10	39.1% (9)
	>10	60.9% (14)
FTND	0–8	5.0 (2.00)
Age started smoking, years	8–44 years	17.96 (6.609)
Last quit attempt	Never	13% (3)
	Past year	34.7% (8)
	Greater than 1 year ago	52.2% (12)
Self-efficacy for quitting	0–10	6.35 (2.740)
Motivation for quitting	1–10	7.91 (2.521)
Delay discounting rates of	$100	−3.449 (2.781)
	$1,000	−3.948 (2.707)

*Unpartnered, single, divorced, separated, widowed; Partnered, married, partnered, or living with significant other. FTND, Fagerström Test for Nicotine Dependence.*

Engagement and retention were high; 98.35% of rTMS sessions were completed and 78.30% (*n* = 18) completed the final outcome assessment (see Supplementary Table 2).

### Latency to Relapse

**FIGURE 1:**
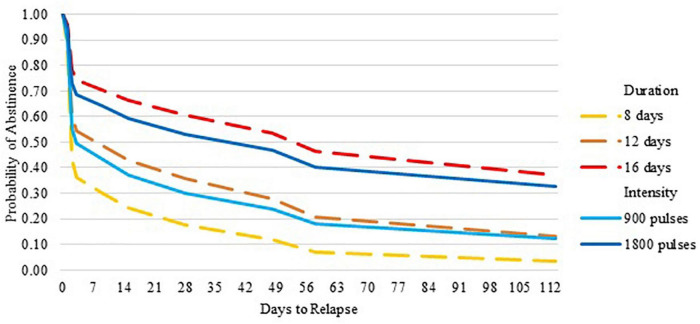
Probability of abstinence over 6 months by duration and intensity of repetitive transcranial magnetic stimulation.

Although the standard deviations and interquartile ranges were large, the mean and median latency to relapse increased as Duration and Intensity increased, as hypothesized. Compared to 8 days of stimulation, 12 days showed a medium effect size (Cohen’s d = 0.310) and 16 days showed a large effect size (Cohen’s *d* = 0.741). Increasing Duration from 8 to 16 days significantly reduced the relative risk of relapse [HR 0.29 (0.09, 1.00); *p* = 0.049], such that with 16 days of stimulation the mean days to relapse increased from 17 days to 76 and the median from 2 to 31 days. Increasing Intensity from 900 to 1,800 pulses per day approached a medium effect size (Cohen’s *d* = 0.381). Increasing Intensity from 900 pulses per day to 1800 pulses reduced the relative risk of relapse [HR 0.53 (0.17, 1.66); *p* = 0.28], such that with 1,800 pulses the mean days to relapse increased from 26 to 64 and the median from 2 to 28 days (see Supplementary Table 3 and Figure 1).

### Point Prevalence Abstinence

Using both ITT and CCA analysis, the proportion of participants abstinent from smoking consistently increased as Duration and Intensity increased. Logistic regressions revealed that increasing Duration from 8 to 12 days and from 8 to 16 days increased the odds of abstinence 4 and 7–8 fold, respectively. Increasing Intensity doubled the odds of long-term abstinence. Nevertheless, the confidence intervals were quite large. Differences were not statistically significant (Duration ITT: χ^2^ = 3.260, *p* = 0.196, *R*^2^ = 0.187; CCA: χ^2^ = 2.885, *p* = 0.236, *R*^2^ = 0.178) (Intensity ITT: χ^2^ = 0.712, *p* = 0.399, *R*^2^ = 0.043; CCA: χ^2^ = 0.421, *p* = 0.516, *R*^2^ = 0.028) (see Supplementary Table 4).

### Participant Burden

MANOVAs revealed small effect sizes for both Duration and Intensity on the total PeRBA score (Duration: *F* = 0.376, *p* = 695, η^2^ = 0.059 and Intensity: *F* = 0.008, *p* = 0.930, η^2^ = 0.001). Similarly, GEE revealed small effect sizes for Duration and Intensity on total PeRBA scores (Duration: χ^2^ = 1.921, *p* = 0.383, Φ = 0.289, Φ^2^ = 0.084 and Intensity: χ^2^ = 0.901, *p* = 0.343, Φ = 0.198, Φ^2^ = 0.039). Increasing Duration or Intensity did not increase research burden and the scores were in the lower range (possible range is 21–105) (see Supplementary Table 5).

### Delay Discounting

**FIGURE 2:**
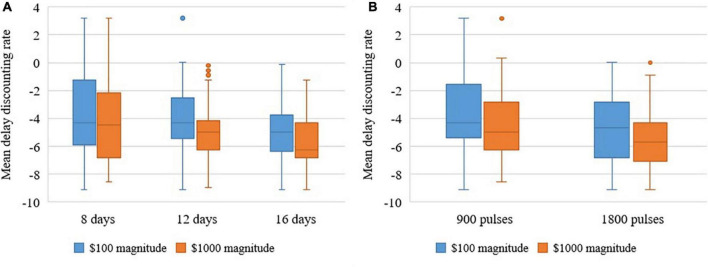
**(A)** Repeated measures analysis of variance shows that increasing duration decreases delay discounting rates of $100 and $1000 overall over 6 months. **(B)** Repeated measures analysis of variance shows that increasing intensity decreases delay discounting rates of $100 and $1000 overall over 6 months.

MANOVAs revealed large effect sizes for Duration (between 8, 12, and 16 days) for the $100 and $1000 magnitudes and these differences were statistically significant ($100: *F* = 4.500, *p* = 0.035, η^2^ = 0.429; and $1,000 *F* = 5.657, *p* = 0.019, η^2^ = 0.485). See Figure 2A the difference for Intensity (between 900 and 1,800 pulses per day) was in the expected direction, with small effect size, and not statistically significant ($100 *F* = 0.083, *p* = 0.779, η^2^ = 0.007; and $1000 *F* = 0.023, *p* = 0.883, η^2^ = 0.002) (see Figure 2B).

**FIGURE 3:**
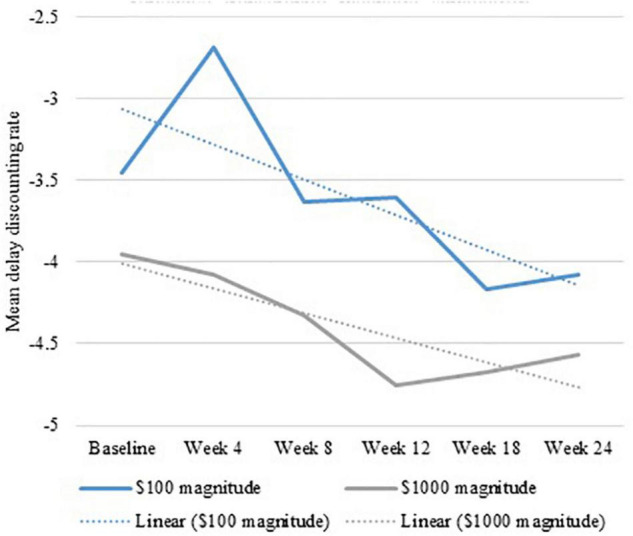
Generalized estimating equation show an overall decrease in delay discounting rate over 6 months of repetitive transcranial magnetic stimulation.

GEE revealed an overall decrease in delay discounting rate over time (see Figure 3) for Duration but not Intensity. Overall, large effect sizes were found for Duration ($100: χ^2^ = 16.008, *p* < 0.001, Φ = 0.834, Φ^2^ = 0.696; and $1,000: χ^2^ = 19.042, *p* < 0.001, Φ = 0.909, Φ^2^ = 0.827). Participants who received 16 days of rTMS had a more robust change in $100 and $1,000 magnitudes across time compared to those receiving 8 days of rTMS. The difference between 900 and 1,800 pulses per day was in the expected direction, with small effects size, and not statistically significant ($100: χ^2^ = 1.187, *p* = 0.28, Φ = 0.227, Φ^2^ = 0.051; and $1,000: χ^2^ = 2.161, *p* = 0.14, Φ = 0.307, Φ^2^ = 0.094) (see Supplementary Table 5).

## Discussion

Using multiple outcomes and statistical approaches, these findings provide consistent preliminary support for the primary smoking cessation hypotheses. Greater Duration and Intensity had greater effects on increasing latency to relapse, improving abstinence rates, and decreasing delay discounting rates. Findings also provided support, though weak, for the hypothesis that increased Duration and Intensity also increase participant research burden. Finally, these findings suggest that the therapeutic target, delay discounting, was robustly engaged and demonstrated the predicted concurrent effects on delay discounting and efficacy outcomes.

These findings suggest that prior to the onset of the COVID-19 pandemic, engagement was sufficiently high among participants who received higher doses of rTMS in terms of Duration and Intensity to support larger efficacy trials. In this sample, 98% of the rTMS sessions were completed, 78% completed the final outcome assessment, and at least some daily cigarette use was collected for all participants. Most participants were of lower income, were diverse in terms of employment, and over 30% identified as racial and/or ethnic minorities suggesting that rTMS studies are able to attract racially and socioeconomically diverse cigarette smokers.

Although increasing both Duration and Intensity increased effect sizes across multiple outcomes, it appears that the number of days of stimulation might have a larger impact on outcomes than the number of pulses per day. This suggests that effects of rTMS on smoking cessation are cumulative and might require time to produce changes in behavior. Future studies with larger sample sizes, should examine whether Duration and Intensity interact to produce higher effect sizes.

Although clearly not conclusive, the impact of increasing Duration and Intensity on perceived research burden was less than expected. We speculate these findings might be an artifact of including only participants who received active stimulation because they were more likely to receive benefits of cessation, which might have outweighed the greater requirements. Future research will need to examine differences in perceived research burden between participants who received active and sham stimulation. Nonetheless, these findings suggest that the burden of participating in this study was not strongly linked with the actual number of rTMS sessions required. Future research will examine whether participants who reached study end after the onset of the pandemic experienced greater participant burden.

The parent study is ongoing and expected to meet modified accrual objectives in 2022. Findings from this study will provide a pre-pandemic comparison for the data collected during the pandemic. Reporting on pre-pandemic findings is important because the pandemic created an environment in which possible biases and confounds potentially impact outcomes. Pre- and post-pandemic comparisons can inform interpretations about biases and confounds should pre- and post-pandemic findings differ.

Finally, future research needs to examine the potential long-term neural adaptations from multiple sessions of rTMS. Although an isolated finding, one study reported a reflection effect, whereby one session of rTMS decreased DD of monetary gains, but also increased DD of monetary losses, a potentially negative finding (Sheffer et al., 2013). Therefore, future research should incorporate the examination of long-term paradoxical or counter therapeutic effects.

The strengths of this study include factorial design in which each participant is exposed to a level of each factor, allowing for the efficient examination of the main effects for Duration and Intensity in one study, eliminating confounds associated with systematic differences among pilot studies using different doses. This design also provides an estimate of the main effects of each factor in the presence of the other factor. Nonetheless, these findings are limited by a small sample size and lack of sham control comparisons. We did not include participants who received sham in this preliminary analysis for multiple reasons. Including sham participants would have doubled the number of cells and comparisons. In addition to the small number of participants who reached study end prior to the onset of the pandemic, the parent study randomized participants to active or sham in a 3–1 ratio. Many of the cells were simply too small to feasibly compare Duration and Intensity when the sham was included. Although including participants who smoke from 6 to 25 cigarettes per day might introduce uncontrolled variability, this limitation is tempered by permuted block randomization stratified by nicotine dependence level. Finally, all participants were motivated to quit based on the inclusion criteria, which limits generalizability of the results to treatment seeking individuals.

## Conclusion

These findings provide preliminary support for targeting delay discounting as a therapeutic target for smoking cessation with rTMS. Greater Duration and Intensity of rTMS appear to have greater effects on delay discounting rates and multiple indicators of abstinence, with a small effect on participant burden. Findings provide a pre-pandemic comparison for the data collected during the pandemic and a basis to examine possible biases and confounds created by the COVID-19 pandemic in the parent study.

## Data Availability Statement

The data analyzed in this study is subject to the following licenses/restrictions: All data, and research materials will be available upon completion of the associated clinical trial with the appropriate permissions (clinical trial identifier: NCT03865472). Requests to access these datasets should be directed to the corresponding author or Clinicaltrials.gov.

## Ethics Statement

The studies involving human participants were reviewed and approved by the Institutional Review Board of Roswell Park Comprehensive Cancer Center (#I-65718). The patients/participants provided their written informed consent to participate in this study.

## Author Contributions

AS, EC, MM, CH, WB, and CS: conceptualization. AS, AL, CR, RA, AB, LB, DV, HT, MM, and CS: methodology. AS, EC, and CS: formal analysis. MM, AL, CR, RA, AB, LB, DV, HT, MM, and CS: investigation. CS: resources, supervision, and funding acquisition. AS, EC, and AL: data curation. AS and CS: writing—original draft preparation. AS, EC, MM, CH, AL, CR, RA, AB, LB, DV, HT, MM, WB, and CE: writing—review and editing. AS: visualization. AL and CS: project administration. All authors have read and agreed to the published version of the manuscript.

## Conflict of Interest

MM had provided expert testimony on the health effects of smoking in lawsuits filed against the tobacco industry. He has also received research support from Pizer, Inc., for an on-going clinical trial of smoking cessation, and has previously served on external advisory panels sponsored by Pfizer to promote smoking cessation in clinical settings. The remaining authors declare that the research was conducted in the absence of any commercial or financial relationships that could be construed as a potential conflict of interest.

## Publisher’s Note

All claims expressed in this article are solely those of the authors and do not necessarily represent those of their affiliated organizations, or those of the publisher, the editors and the reviewers. Any product that may be evaluated in this article, or claim that may be made by its manufacturer, is not guaranteed or endorsed by the publisher.
