# Supplementary material for: Transcranial Magnetic Stimulation for Long-Term Smoking Cessation: Preliminary Examination of Delay Discounting as a Therapeutic Target and the Effects of Intensity and Duration
Source: Front Hum Neurosci. 2022 Jul 5;16:920383. doi: 10.3389/fnhum.2022.920383 (PMC9300313; doi:10.3389/fnhum.2022.920383)
Supplement: Supplementary file 1 [file Data_Sheet_1.pdf]

Supplementary material

Supplemental Table 1. Participant level of motivation to quit and impulsivity levels throughout the study

Variable	Baseline	Week 4	Week 8	Week 12	Week 16	Week 24
Motivation to quit M (SD)	7.91 (2.52)	9.32 (1.21)	9.42 (1.26)	8.90 (1.68)	9.22 (1.16)	8.56 (1.69)
BIS score M (SD)	57.52 (8.02)	57.14 (7.54)	57.32 (8.98)	56.80 (10.69)	57.11 (9.68)	56.44 (9.95)

Note: BIS= Barratt Impulsiveness Scale

Supplemental Table 2. Participant engagement and retention

		Duration		
		**8 days in 2 weeks**	**12 days in 3 weeks**	**16 days in 4 weeks**
**Intensity**	**900 pulses per day (1 session per day)**	n=7<br>100% of rTMS sessions completed<br>85.71% (n=6) completed final outcome assessment	n=5<br>100% of rTMS sessions completed<br>60% (n=3) completed final outcome assessment	n=4<br>100% of rTMS sessions completed<br>100% (n=4) completed final outcome assessment
	**1800 pulses per day (2 sessions per day)**	n=2<br>81.25% of rTMS sessions completed<br>50% (n=1) completed final outcome assessment	n=1<br>100% of rTMS sessions completed<br>100% (n=1) completed final outcome assessment	n=4<br>100% of rTMS sessions completed<br>75% (n=3) completed final outcome assessment

Supplemental Table 3. Relative risk of relapse tended to decrease as duration and intensity of rTMS increased

Predictor	/Categories	Mean days until relapse (SD)	Median days until relapse (IQR)	Cohen's d	B (SE)	Hazard Ratios (95% CI) and p value	χ2,p
Duration	8 days	17 (37.04)	2 (1.5-15)	comparison group			χ2=4.27, p=.118
	12 days[a]	16 (17.22)	12 (2-27)	.31	-.52 (.63)	.597 (.17, 2.05); p=.412	
	16 days[a]	76.63 (89.27)	30.50 (2-182)	.74	-1.24 (.63)	.291 (.09, .99); p=.049	
Intensity	900 pulses	26 (51.11)	2 (2-18.75)	comparison group			χ2=1.22, p=.269
	1800 pulses[b]	63.71 (82.60)	28 (2-182)	.38	-.64 (.58)	.530 (.17, 1.66); p=.276	

[a]Comparison group is 8 days; [b]Comparison group is 900 pulses per day;
SD= Standard deviation; IQR=Interquartile range; SE=Standard error; CI=Confidence intervals

Supplemental Table 4. Six month point prevalence abstinence rates increased as duration and intensity of rTMS increased

Predictor /Categories		Odds Ratios (95%CI)	Proportion abstinent
Duration of rTMS	8 days	comparison group	12.5% abstinent (CCA)
			11.1% abstinent (ITT)
	12 days[a]	4.667 (.297, 73.384)	40% abstinent (CCA)
		4.000 (.273, 58.562)	33.3% abstinent (ITT)
	16 days[a]	7.000 (.568, 86.321)	50% abstinent (CCA)
		8.000 (.658, 97.311)	50% abstinent (ITT)
Intensity	900 pulses	comparison group	28.6% abstinent (CCA)
			25% abstinent (ITT)
	1800 pulses[b]	1.875 (.282,12.455)	42.9% abstinent (CCA)
		2.250 (.345,14.694)	42.9% abstinent (ITT)

[a]Comparison group is 8 days of rTMS; [b]Comparison group is 900 pulses per day of rTMS
CCA = Complete case analysis, ITT = intention to treat using right censored analysis

Supplemental Table 5. Generalized Estimating Equations for Duration and Intensity of rTMS predicting changes in delayed discounting outcome measures across baseline and outcome assessments

**Outcome**	**Duration/ Intensity of rTMS**	**Overall Est. Means**	**B**	**SE**	**CI**		**p**
					**lower**	**upper**	
Delay discounting rates of $100**	8 days	-2.49	Comparison group				
	12 days	-2.95	-.47	.88	-2.18	1.25	.60
	16 days	-5.12	-2.63	.70	-4.01	-1.25	.00
Delay discounting rates of $1000**	8 days	-2.94	Comparison group				
	12 days	-4.18	-1.24	.89	-2.97	.50	.16
	16 days	-5.99	-3.06	.74	-4.50	-1.61	.00
PeRBA	8 days	29.28	Comparison group				
	12 days	28.67	-.62	2.32	-5.16	3.93	.79
	16 days	33.14	3.85	2.97	-1.97	9.68	.19
Delay discounting rates of $100	900 pulses	-3.28	Comparison group				
	1800 pulses	-4.29	-1.01	.93	-2.83	.81	.28
Delay discounting rates of $1000	900 pulses	-4.00	Comparison group				
	1800 pulses	-5.31	-1.32	.90	-3.09	.44	.14
PeRBA	900 pulses	29.72	Comparison group				
	1800 pulses	32.62	2.90	3.05	-3.09	8.88	.34

** p < .001; Comparison group is 8 days; Note: Est. means are estimated means across all time points
